# DropletQC: improved identification of empty droplets and damaged cells in single-cell RNA-seq data

**DOI:** 10.1186/s13059-021-02547-0

**Published:** 2021-12-02

**Authors:** Walter Muskovic, Joseph E. Powell

**Affiliations:** 1 Garvan Weizmann Centre for Cellular Genomics, Garvan Institute of Medical Research, The Kinghorn Cancer Centre, Darlinghurst, NSW 2010 Australia; 2 UNSW Cellular Genomics Futures Institute, University of New South Wales, Sydney, NSW 2052 Australia

## Abstract

**Background:**

Advances in droplet-based single-cell RNA-sequencing (scRNA-seq) have dramatically increased throughput, allowing tens of thousands of cells to be routinely sequenced in a single experiment. In addition to cells, droplets capture cell-free “ambient” RNA predominantly caused by lysis of cells during sample preparation. Samples with high ambient RNA concentration can create challenges in accurately distinguishing cell-containing droplets and droplets containing ambient RNA. Current methods to separate these groups often retain a significant number of droplets that do not contain cells or empty droplets. Additionally, there are currently no methods available to detect droplets containing damaged cells, which comprise partially lysed cells, the original source of the ambient RNA.

**Results:**

Here, we describe DropletQC, a new method that is able to detect empty droplets, damaged, and intact cells, and accurately distinguish them from one another. This approach is based on a novel quality control metric, the nuclear fraction, which quantifies for each droplet the fraction of RNA originating from unspliced, nuclear pre-mRNA. We demonstrate how DropletQC provides a powerful extension to existing computational methods for identifying empty droplets such as EmptyDrops.

**Conclusions:**

We implement DropletQC as an R package, which can be easily integrated into existing single-cell analysis workflows.

**Supplementary Information:**

The online version contains supplementary material available at 10.1186/s13059-021-02547-0.

## Main text

Droplet-based single-cell RNA-sequencing (scRNA-seq) methods utilize microfluidics to encapsulate individual cells in nanoliter droplet emulsions, a technique that has dramatically increased throughput compared to plate-based protocols [1]. While encapsulating cells, droplets also capture cell-free ambient RNA, a complex mixture of transcripts released from damaged, stressed, and dying cells, often exacerbated during dissociation of solid tissues. This ambient RNA creates challenges for downstream analyses and the biological interpretation of results as most analysis methods are based on the assumption that a droplet contains RNA from a single cell. To combat this problem, several methods have been developed to estimate and remove its contribution to gene expression [2–4].

High levels of ambient RNA also create challenges in accurately identifying cell-containing droplets. This is a particular problem for data generated from solid tissues, where more fragile cells are more likely to become damaged during dissociation and contribute to ambient RNA. We thus have three scenarios that need to be differentiated: empty droplets containing high concentrations of ambient RNA, droplets containing damaged cells, and droplets containing cells with limited ambient RNA. Using cut-offs based on the total number of RNA fragments assigned to each droplet, such as those originally proposed by Macosko et al*.* [5] and Zheng et al*.* [6], risks both including empty droplets and excluding small cells with below-average RNA content. The *EmptyDrops* method [7] addresses this issue through a more sophisticated approach, calculating the profile of the ambient RNA pool and testing each barcode for significant deviations from this profile. A favored alternative to simple UMI cut-offs, this method has been integrated as the default cell-calling algorithm in the widely used 10x Genomics *Cell Ranger* pipeline [6]. However, cell-free droplets with high ambient RNA concentration and damaged cells are still retained by this method.

**Fig. 1:**
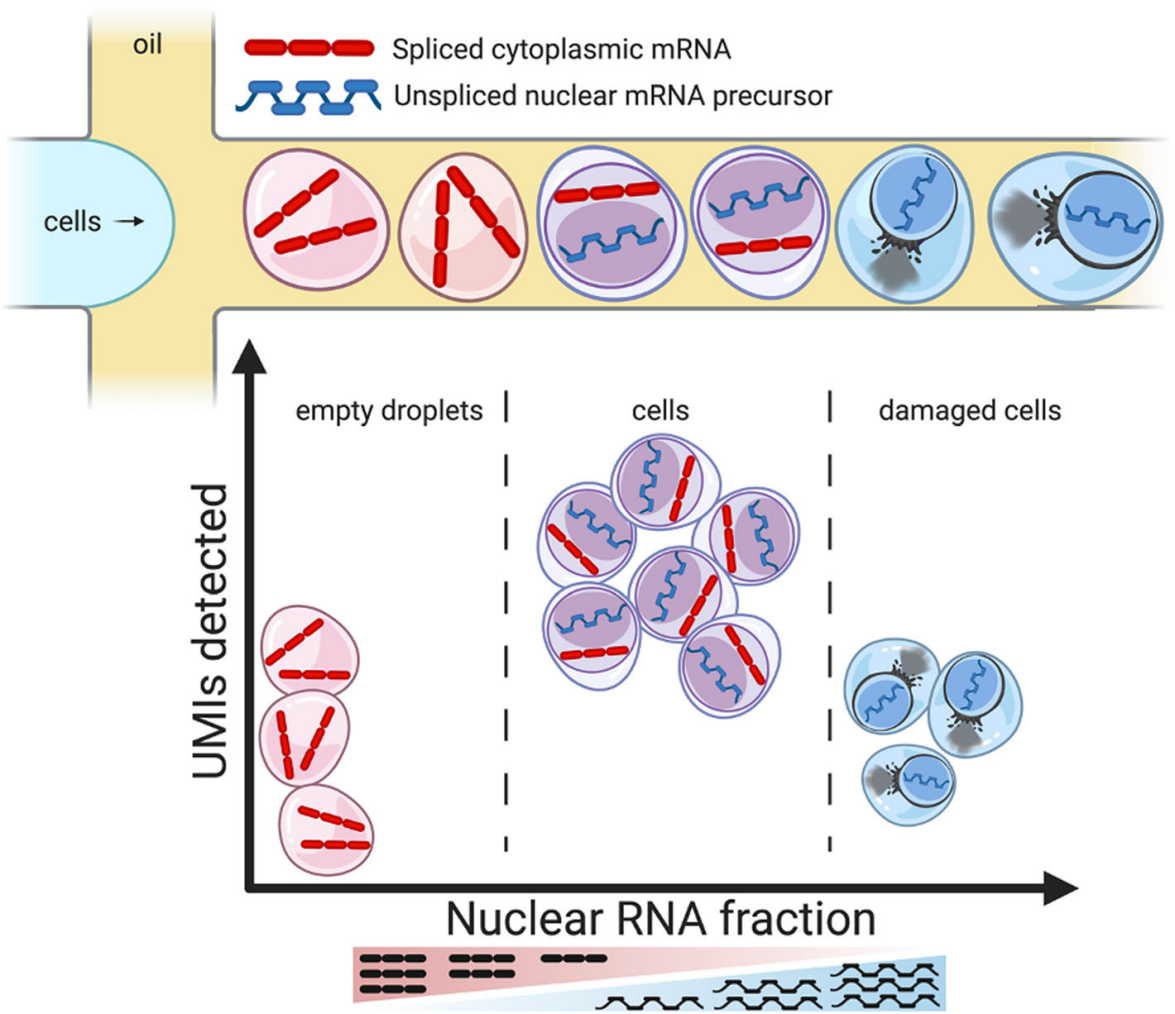
Illustration of how the nuclear fraction, in combination with the library size of each droplet, can be used to separate the populations of empty droplets, intact cells, and damaged cells

Here, we present *DropletQC*, a new method that is able to simultaneously improve the detection of cell free droplets and droplets containing damaged cells. Taking advantage of the observation that unspliced and spliced mRNAs can be distinguished in common scRNA-seq protocols [8], we develop a novel metric: the nuclear fraction. The nuclear fraction quantifies, for each droplet, the proportion of RNA originating from unspliced pre-mRNA. Ambient RNA consists predominantly of mature cytoplasmic mRNA. This may arise as RNA is released from damaged cells in which the nuclear envelope remains intact, or capped and polyadenylated transcripts may be more stable in the extracellular environment (Fig. 1). Regardless, droplets that contain only ambient RNA have a low nuclear fraction compared to droplets containing cells (Additional file 1: Figure S1). In contrast, damaged cells due to the depletion of cytoplasmic RNA will have a higher nuclear fraction compared to intact cells. By using the nuclear fraction score in combination with UMIs per droplet, we are able to accurately distinguish between empty droplets, damaged cells, and intact cells.

**Fig. 2:**
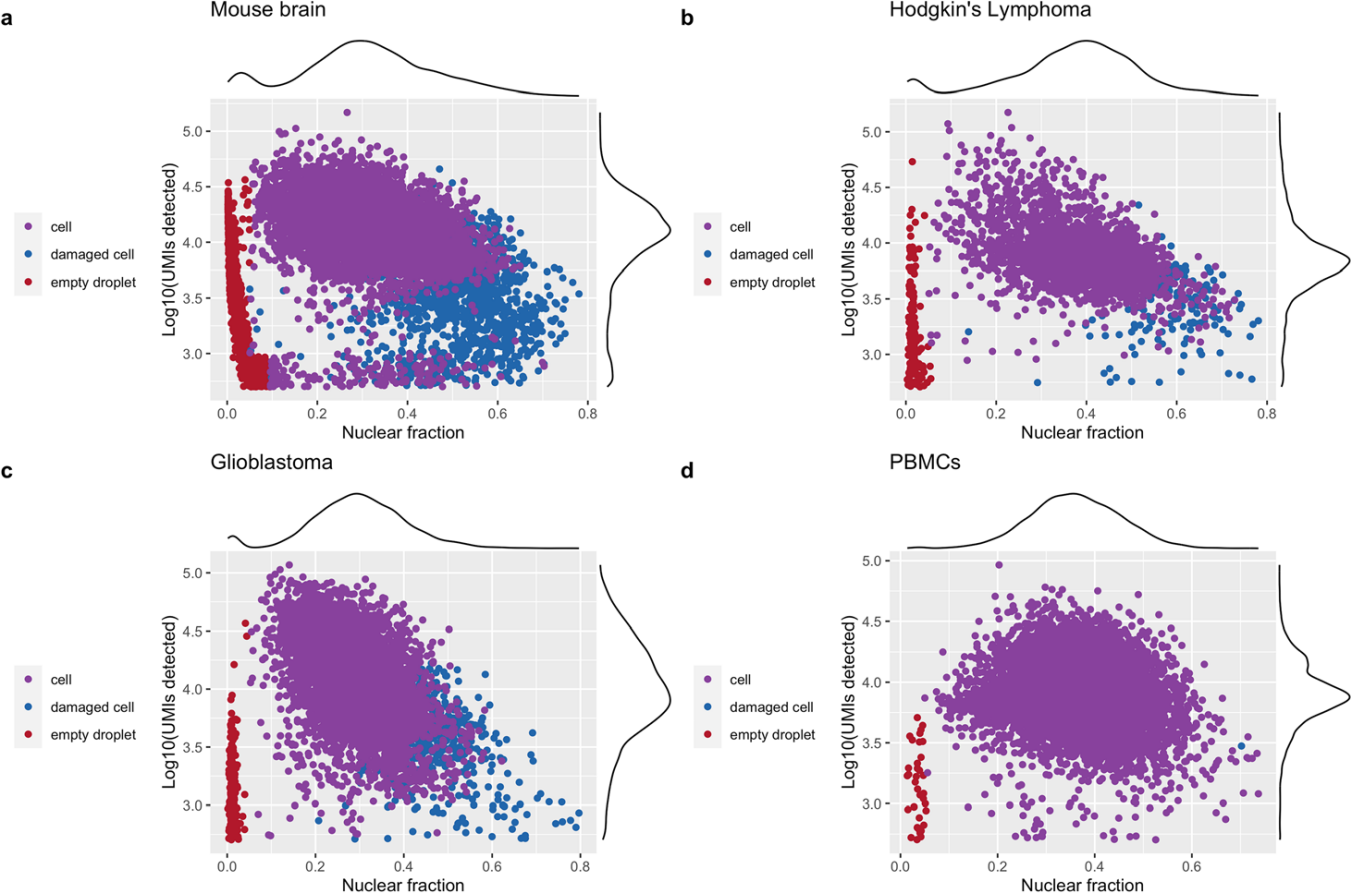
*DropletQC* identifies empty droplets and damaged cells in four heterogeneous scRNA-seq datasets. Total UMI counts (*y*-axis) and nuclear fraction scores (*x*-axis) are shown for each cell, with colors representing the status of each cell assigned by *DropletQC*. Empty droplets contain less RNA than cells and a higher fraction of cytoplasmic RNA (low nuclear fraction score). Damaged cells contain less RNA than intact cells and a higher proportion of unspliced RNA fragments (high nuclear fraction score)

To assess the ability of *DropletQC* to identify both empty droplets and droplets containing damaged cells, we applied it to four independent scRNA-seq datasets; embryonic mouse brain, glioblastoma tumor, peripheral blood mononuclear cells (PBMCs), and Hodgkin’s lymphoma tumor. To determine whether *DropletQC* could identify empty droplets missed by current methods, barcodes were filtered using 10x Genomics *Cell Ranger 6.1.1* [6], *CellBender* [9], *EmptyNN* [10], or *EmptyDrops* as implemented in *DropletUtils* [7]. For all methods tested, *DropletQC* identified additional cell free droplets (Additional file 1: Figure S2). These droplets are identified by *DropletQC* using an automatic threshold based on the distinctly lower nuclear fraction scores exhibited compared to droplets containing cells, which contain a mixture of mature (cytoplasmic) and precursor (nuclear) mRNA (Fig. 2). To validate the identified droplets do not contain intact cells, the levels of two transcripts; *MALAT1* and *NEAT1* were quantified for each droplet. These abundant lncRNAs maintain structural roles in nuclear speckles and paraspeckles respectively and are retained exclusively within the cell nucleus [11]. Droplets identified by DropletQC as cell-free displayed low levels of both transcripts (Additional file 1: Figure S3), indicating the droplets identified as empty do not contain intact cells. Cell free droplets represent 9.5% of mouse brain, 6.0% of Hodgkin’s lymphoma, 4.0% of glioblastoma, and 0.4% of PBMCs retained following filtering with *EmptyDrops* (Additional file 1: Table S1). Cells from dissociated tissue (Fig. 2a–c) contained more empty droplets with high RNA content than PBMCs (Fig. 2d), suggesting ambient RNA may be released from cells damaged during sample preparation.

Following identification of empty droplets, droplets containing damaged cells are identified using expectation maximization and a Gaussian mixture model to separate them from droplets containing intact cells. As both the total UMI count and nuclear fraction scores display distinct distributions for different cell types (Additional file 1: Figure S4), it is necessary to first annotate cell types, so that damaged cells may be identified separately for each. Cells were annotated for each sample using a combination of gene markers and supervised classification with *scPred* [12]. Of the remaining cells, 14.0% of mouse brain, 5.2% of Hodgkin’s lymphoma, 9.8% of glioblastoma tumor cells, and one PBMC cell were identified as damaged cells (Additional file 1: Table S1).

As an additional test of the ability of *DropletQC* to identify damaged cells, we applied the method to data from a recent investigation on the effects of cryopreservation on the transcriptomes of macaque microglia [13]. *DropletQC* revealed an increase in the proportion of damaged cells following cryopreservation from 4.1 to 13.8% (Additional file 1: Figure S5, Table S2). These findings have implications for the suitability of prospectively archiving samples for scRNA-seq studies through cryopreservation and demonstrates the utility of *DropletQC* for similar studies. To validate that the cells identified by *DropletQC* are indeed damaged, we applied the method to data from HEK293 cells stressed with staurosporine and captured in healthy, pro-, and late-apoptotic states [14]. *DropletQC* identified an increase in both the proportion of damaged cells and cell free droplets as cells progressed toward the late-apoptotic state (Additional file 1: Figure S6a-d). HEK293 cells identified as damaged were associated with a higher mitochdondrial gene content (Additional file 1: Figure S6e) a hallmark of damaged and dying cells [15]. Similarly, damaged cells for other datasets were associated with a higher mitochondrial gene content (Additional file 1: Figure S7).

To assess whether *DropletQC* is applicable to commonly used 5′ scRNA-seq protocols, we examined two additional scRNA-seq datasets; glioblastoma tumor 5′ v1 and mouse splenocytes 5′ v2. After filtering with *EmptyDrops*, *DropletQC* identified both empty droplets and damaged cells in both samples (Additional file 1: Figure S8, Table S3). As common protocols capture unspliced intronic sequences [8], we anticipate *DropletQC* will be applicable to most scRNA-seq datasets.

By default, the *DropletQC* software provides a flag for empty droplets and damaged cells, but does not remove them from the dataset. Depending on the biological analyses, damaged cells may retain useful information, and as such, it may be desirable to retain this metadata throughout downstream analyses. In addition, care should be taken that cells are accurately annotated, to avoid confounding distinct cell types as populations of damaged and intact cells. Similarly, cells such as erythrocytes, which contain small amounts of mature mRNA, may be misidentified as empty droplets and can be rescued downstream if desired. We note that intron and exon boundaries should be well-defined in the reference transcriptome for accurate estimation of the nuclear fraction. An option to calculate the nuclear fraction using a user-provided gene annotation file is provided, so that it may be easily recalculated as the quality of a species’ gene annotation is improved.

For samples with large percentages of ambient RNA, some damaged cells and empty droplets may be missed by *DropletQC*. However, these can be identified by their low RNA content (Fig. 2a) and may be easily flagged using a minimum UMI threshold. We recommend that *DropletQC* be used in tandem with a tool such as *EmptyDrops* to prune the majority of cell barcodes, before identifying any remaining cell free droplets or damaged cells. Calculation of the nuclear fraction, identification of empty droplets and damaged cells are implemented as separate functions within the *DropletQC* package.

In summary, we have shown that *DropletQC* is able to successfully identify both empty droplets and damaged cells in data from a range of tissue types.

## Methods

## Nuclear fraction calculation

The *DropletQC* method first calculates the nuclear fraction for each droplet, which is the proportion of RNA fragments that originate from intronic regions. It is calculated as:

$$ {NF}_i=\frac{\Sigma \left({IR}_i\right)}{\Sigma \left({IR}_i+{ER}_i\right)} $$

where *NF*_*i*_ is the nuclear fraction for droplet *i*, *IR*_*i*_ are the reads that map to intronic regions for droplet *i*, and *ER*_*i*_ are those that map to exonic regions. We have implemented two methods to map reads to either intronic or exonic regions. The first takes advantage of region tags, such as those added by 10x Genomics’ *Cell Ranger* count analysis pipeline that identify the region type of each genome-aligned RNA fragment; exonic, intronic, or intergenic. These are efficiently counted using the nuclear_fraction_tags function to calculate a nuclear fraction score for each provided cell barcode. Alternatively, if region tags are missing, our second method assesses aligned reads for overlap with intronic regions using the nuclear_fraction_annotation function in combination with a user-provided gene annotation file. To speed up processing of indexed, coordinate-sorted alignment files, reads are split across a user-specified number of genomic regions to allow parallel computation. The four samples presented in the manuscript were processed with 8 CPUs and 16Gb of RAM with an average processing time of 106 s per 100 million reads using the nuclear_fraction_tags function and 132 s per 100 million reads using the nuclear_fraction_annotation function.

### Identifying empty droplets and damaged cells

Empty droplets are classified as all barcodes that fall below a defined nuclear fraction threshold. To identify a suitable threshold, a kernel density estimate is calculated using the nuclear fraction scores. The first derivative of the estimate is then calculated to identify the local minimum immediately following the first peak, corresponding to the population of empty droplets. If the automatically selected cut-off misidentifies the empty droplet population, two user-adjustable parameters are provided; a nuclear fraction threshold and a total UMI threshold, above which all barcodes are marked as cells.

To identify droplets containing damaged cells, barcodes are assessed separately for each cell type. It is assumed damaged cells have both a lower UMI count and higher nuclear fraction score than undamaged cells. We therefore use a two component (*k*) gaussian mixture model, implemented with *mclust* [16], to classify droplets containing damaged cells:

Eq. 1 $$ P\left(X\ \right|\mu, \sigma, \alpha \Big)={\alpha}_1N\left(X|{\mu}_1,{\sigma}_1^2\right)+{\alpha}_2N\left(X|{\mu}_2,{\sigma}_2^2\right) $$

where *X* is a dataset with *log*_10_(*UMI*) and estimated nuclear fractions for *1-n* droplets of a given cell type. *μ* and *σ*^2^are the mean and variance, and *α* represents the mixing weight of a given component. The initial model parameters are calculated as:

$$ {\mu}_k=\frac{\sum_i^{N_k}{x}_{i,k}}{N_k} $$

$$ {\sigma}_k^2=\frac{\sum_i^{N_k}{\left({x}_{i,k}-{\mu}_k\right)}^2}{N_k} $$

$$ {\alpha}_k=\frac{N_k}{N} $$

where *N*_*k*_is the number of data points in the *k*th component. Following the initialization, we estimate parameters using expectation maximization by asking what is the posterior probability that a droplet (*x*_*i*_) belongs to component *k*_*j*_:

Eq. 2 $$ P\left({x}_i\in {k}_j|{x}_i\right)=\frac{P\left({x}_i|{x}_i\in {k}_j\right)P\left({k}_j\right)}{P\left({x}_i\right)} $$

where,

$$ P\left({x}_i|{x}_i\in {k}_j\right)=N\left({x}_i|{\mu}_{kj},{\sigma}_{kj}^2\right) $$

$$ P\left({k}_j\right)={\alpha}_{kj} $$

$$ P\left({x}_i\right)={\sum}_{k=1}^K{\alpha}_kN\left({x}_i|{\mu}_k,{\sigma}_k^2\right) $$

*N*_*k*_in the initial component parameters are replaced with the posterior probability and recalculated, with these steps repeated until convergence determined using the Bayesian information criterion. This model identifies the minimum separation required between the identified distributions for a population of droplets to be marked as damaged. We then label droplets as containing a damaged cell based on a higher mean nuclear fraction and lower mean UMI than the cell population, a mean nuclear fraction greater than the cell population mean by a user-adjustable amount (default 0.15), and a mean UMI count lower than the cell population (default 50%).

### Data

### Cell filtering and annotation

For the mouse brain, Hodgkin’s lymphoma, glioblastoma, and PBMC samples presented in Fig. 2, prior to calculating the nuclear fraction score, all cell barcodes were assessed for a significant deviation from the ambient RNA expression pattern using the *EmptyDrops* method implemented in *DropletUtils* [7]. The lower bound on the total UMI count used to identify empty droplets was increased from 100 to 500 and all other parameters were left at their default values. Barcodes below a false discovery rate threshold of 1% were excluded. Remaining barcodes were additionally filtered for a maximum mitochondrial gene content of 15% to exclude low-quality cells, in line with current best practices when assessing common scRNA-seq quality control metrics. Mouse brain and PBMC cell types were annotated by supervised classification with the *scPred* [12] using annotated PBMC [17], mouse brain [18], and developing mouse brain [19] reference datasets. The glioblastoma sample cell types were identified using cell-type specific gene markers for oligodendrocytes (*MAG*, *MOG*, *MBP*), microglia/macrophages (*C1QA*, *AIF1*, *LAPTM5*), T cells (*CD2*, *CD3D*, *CD3E*), and endothelial cells (*CD34*, *ESAM*, *APOLD1*) [20–23]. Hodgkin’s lymphoma cell types were classified using marker genes for B cells (*MS4A1*), macrophages (*CD68*, *IDO1*), plasmacytoid dendritic cells (*CLEC4C*, *NRP1*), erythrocytes (*HBB*, *HBA1*, *HBA2*), cytotoxic T cells (*GZMA*, *GZMK*, *IFNG*), regulatory T cells (*FOXP3*, *IL2RA*, *IKZF2*), T helper cells (*CXCL13*, *PDCD1*, *FABP5*), naïve T cells (*CCR7*, *IL7R*, *LEF1*), progenitor (*CD34*), and mast cells (*TPSAB1*, *TPSB2*, *KIT*) [24, 25].

### Method comparison

*CellBender*’s *remove-background* function was run with the number of epochs set to 150 and fpr of 0.01, as per the default parameters. The total-droplets-included argument was set to 20,000 and the expected-cells argument to 10,000 for the mouse brain and PBMC datasets and 5000 for the glioblastoma and Hodgkin’s lymphoma samples. *EmptyNN* was run with the number of k-folds set to 10, for 10 iterations. The UMI counts threshold was set to the default value of 100, as well as a value of 500. *EmptyDrops* was run with default parameters and the lower bound on the total UMI count set to the default value of 100, as well as 500. Barcodes below a false discovery rate threshold of 1% were excluded as empty droplets. To provide a fair comparison with existing practices for assessing QC metrics, cells were additionally filtered with a mitochondrial gene content threshold of 15% before being assessed with *DropletQC*.

## Supplementary Information

**Additional file 1.** A document containing supplementary figures 1-8 and supplementary tables 1-3 referenced in the main text of the manuscript

**Additional file 2.** Review history

## Data Availability

The four 3′ single-cell gene expression datasets presented in Fig. 2 of the manuscript as well as the two 5′ datasets presented in Additional file 1: Figure S8 are made publicly available through the 10x Genomics website: https://support.10xgenomics.com/single-cell-gene-expression/datasets [26–31]. The macaque microglia expression data is available from the NCBI GEO database, under accession GSE162663 [32]. The staurosporine-treated HEK293 cell dataset is available from the ENA repository with the study accession number PRJEB33078 [33]. All of the code used to produce the analyses and figures presented in the manuscript, along with links to individual datasets, are available through GitHub at https://github.com/powellgenomicslab/dropletQC_paper [34] under the MIT license and on zenodo at doi:10.5281/zenodo.5708997 [35]. *DropletQC* is available as an *R* package at https://github.com/powellgenomicslab/DropletQC [36] under the MIT license and on zenodo at doi:10.5281/zenodo.5708994 [37].

## References

[1] Svensson V, Vento-Tormo R, Teichmann SA. Exponential scaling of single-cell RNA-seq in the past decade. Nat Protoc. Nature Publishing Group; 2018. 599–604. 10.1038/nprot.2017.149 29494575

[2] Yang S, Corbett SE, Koga Y, Wang Z, Johnson WE, Yajima M, et al. Decontamination of ambient RNA in single-cell RNA-seq with DecontX. Genome Biol. BioMed Central Ltd.; 2020;21:57. 10.1186/s13059-020-1950-6 PMC7059395 32138770

[3] Young MD, Behjati S (2020). SoupX removes ambient RNA contamination from droplet-based single-cell RNA sequencing data. Gigascience.

[4] Heaton H, Talman AM, Knights A, Imaz M, Gaffney DJ, Durbin R, Hemberg M, Lawniczak MKN (2020). Souporcell: robust clustering of single-cell RNA-seq data by genotype without reference genotypes. Nat Methods.

[5] Macosko EZ, Basu A, Satija R, Nemesh J, Shekhar K, Goldman M, et al. Highly parallel genome-wide expression profiling of individual cells using nanoliter droplets. Cell. Cell Press; 2015;161:1202–1214. 10.1016/j.cell.2015.05.002 PMC4481139 26000488

[6] Zheng GXY, Terry JM, Belgrader P, Ryvkin P, Bent ZW, Wilson R, et al. Massively parallel digital transcriptional profiling of single cells. Nat Commun. Nature Publishing Group; 2017;8:1–12. 10.1038/ncomms14049 PMC5241818 28091601

[7] Lun ATL, Riesenfeld S, Andrews T, Dao TP, Gomes T, Marioni JC. EmptyDrops: distinguishing cells from empty droplets in droplet-based single-cell RNA sequencing data. Genome Biol. BioMed Central Ltd.; 2019;20:63. 10.1186/s13059-019-1662-y PMC6431044 30902100

[8] La Manno G, Soldatov R, Zeisel A, Braun E, Hochgerner H, Petukhov V, et al. RNA velocity of single cells. Nature. Nature Publishing Group; 2018;560:494–498. 10.1038/s41586-018-0414-6 PMC6130801 30089906

[9] Fleming SJ, Marioni JC, Babadi M. CellBender remove-background: a deep generative model for unsupervised removal of background noise from scRNA-seq datasets. bioRxiv. Cold Spring Harbor Laboratory; 2019;791699.

[10] Yan F, Zhao Z, Simon LM. EmptyNN: a neural network based on positive and unlabeled learning to remove cell-free droplets and recover lost cells in scRNA-seq data. Patterns. Elsevier; 2021;2:100311. 10.1016/j.patter.2021.100311 PMC8369248 34430929

[11] Hutchinson JN, Ensminger AW, Clemson CM, Lynch CR, Lawrence JB, Chess A. A screen for nuclear transcripts identifies two linked noncoding RNAs associated with SC35 splicing domains. BMC Genomics. BioMed Central; 2007;8:1–16. 10.1186/1471-2164-8-39 PMC1800850 17270048

[12] Alquicira-Hernandez J, Sathe A, Ji HP, Nguyen Q, Powell JE (2019). scPred: accurate supervised method for cell-type classification from single-cell RNA-seq data. Genome Biol.

[13] Morsey B, Niu M, Dyavar SR, Fletcher CV, Lamberty BG, Emanuel K, et al. Cryopreservation of microglia enables single-cell RNA sequencing with minimal effects on disease-related gene expression patterns. iScience. Elsevier BV; 2021;24:102357. 10.1016/j.isci.2021.102357 PMC8044433 33870145

[14] Ordoñez-Rueda D, Baying B, Pavlinic D, Alessandri L, Yeboah Y, Landry JJM, et al. Apoptotic cell exclusion and bias-free single-cell selection are important quality control requirements for successful single-cell sequencing applications. Cytom Part A. John Wiley & Sons, Ltd; 2020;97:156–167. 10.1002/cyto.a.23898 31603610

[15] Ilicic T, Kim JK, Kolodziejczyk AA, Bagger FO, McCarthy DJ, Marioni JC, et al. Classification of low quality cells from single-cell RNA-seq data. Genome Biol. BioMed Central; 2016;17:1–15. 10.1186/s13059-016-0888-1 PMC4758103 26887813

[16] Scrucca L, Fop M, Murphy TB, Raftery AE. Mclust 5: clustering, classification and density estimation using Gaussian finite mixture models. R J. Technische Universitaet Wien; 2016;8:289–317. PMC5096736 27818791

[17] Senabouth A, Andersen S, Shi Q, Shi L, Jiang F, Zhang W, et al. Comparative performance of the BGI and Illumina sequencing technology for single-cell RNA-sequencing. NAR Genomics Bioinforma. Oxford University Press (OUP); 2020;2(2):lqaa034. 10.1093/nargab/lqaa034. 10.1093/nargab/lqaa034 PMC7671348 33575589

[18] Yao Z, Nguyen TN, van Velthoven CTJ, Goldy J, Sedeno-Cortes AE, Baftizadeh F (2021). A taxonomy of transcriptomic cell types across the isocortex and hippocampal formation. Cell.

[19] Rosenberg AB, Roco CM, Muscat RA, Kuchina A, Sample P, Yao Z (2018). Single-cell profiling of the developing mouse brain and spinal cord with split-pool barcoding. Science (80- ). Am Assoc Adv Sci.

[20] Dusart P, Hallström BM, Renné T, Odeberg J, Uhlén M, Butler LM. A systems-based map of human brain cell-type enriched genes and malignancy-associated endothelial changes. Cell Rep. Elsevier B.V.; 2019;29:1690–706.e4. 10.1016/j.celrep.2019.09.088 31693905

[21] Neftel C, Laffy J, Filbin MG, Hara T, Shore ME, Rahme GJ, et al. An integrative model of cellular states, plasticity, and genetics for glioblastoma. Cell. Elsevier; 2019;178:835–49.e21. 10.1016/j.cell.2019.06.024 PMC6703186 31327527

[22] Couturier CP, Ayyadhury S, Le PU, Nadaf J, Monlong J, Riva G, et al. Single-cell RNA-seq reveals that glioblastoma recapitulates a normal neurodevelopmental hierarchy. Nat Commun. Nature Publishing Group; 2020;11:3406. 10.1038/s41467-020-17186-5 PMC7343844 32641768

[23] Wang L, Babikir H, Müller S, Yagnik G, Shamardani K, Catalan F (2019). The phenotypes of proliferating glioblastoma cells reside on a single axis of variation. Cancer Discov.

[24] Aoki T, Chong LC, Takata K, Milne K, Hav M, Colombo A, et al. Single-cell transcriptome analysis reveals disease-defining t-cell subsets in the tumor microenvironment of classic hodgkin lymphoma. Cancer Discov. American Association for Cancer Research Inc.; 2020;10:406–421. 10.1158/2159-8290.CD-19-0680 31857391

[25] Schafflick D, Xu CA, Hartlehnert M, Cole M, Schulte-Mecklenbeck A, Lautwein T, et al. Integrated single cell analysis of blood and cerebrospinal fluid leukocytes in multiple sclerosis. Nat Commun. Nature Research; 2020;11:1–14. 10.1038/s41467-019-14118-w PMC6959356 31937773

[26] Human glioblastoma multiforme (v3), single cell gene expression dataset by Cell Ranger 4.0.0, 10x Genomics, https://support.10xgenomics.com/single-cell-gene-expression/datasets. 2021.

[27] 10k Peripheral blood mononuclear cells from a healthy donor, Dual Indexed, single cell gene expression dataset by Cell Ranger 4.0.0, 10x Genomics, https://support.10xgenomics.com/single-cell-gene-expression/datasets. 2021.

[28] 10k Brain Cells from an E18 Mouse (v3), single cell gene expression dataset by Cell Ranger 3.0.0, 10x Genomics, https://support.10xgenomics.com/single-cell-gene-expression/datasets. 2021.

[29] Hodgkin’s lymphoma, dissociated tumor, whole transcriptome, single cell gene expression dataset by Cell Ranger 4.0.0, 10x Genomics, https://support.10xgenomics.com/single-cell-gene-expression/datasets. 2021.

[30] Human glioblastoma multiforme (5’v1), single cell immune profiling dataset by Cell Ranger 4.0.0, 10x Genomics, https://support.10xgenomics.com/single-cell-gene-expression/datasets. 2021.

[31] Mouse splenocytes (5’v2), single cell immune profiling dataset by Cell Ranger 6.0.1, 10x Genomics, https://support.10xgenomics.com/single-cell-gene-expression/datasets. 2021.

[32] Morsey B, Niu M, Dyavar SR, Fletcher CV, Lamberty BG, Emanuel K, et al. Cryopreservation of microglia enables single-cell RNA sequencing with minimal effects on disease-related gene expression patterns. Gene Expression Omnibus. https://www.ncbi.nlm.nih.gov/geo/query/acc.cgi?acc=GSE162663. 2021. 10.1016/j.isci.2021.102357 PMC8044433 33870145

[33] Ordoñez-Rueda D, Baying B, Pavlinic D, Alessandri L, Yeboah Y, Landry JJM, et al. Apoptotic cell exclusion and bias-free single-cell selection are important QC requirements for successful single-cell sequencing applications. European Nucleotide Archive, https://www.ebi.ac.uk/ena/browser/view/PRJEB33078. 2020. 10.1002/cyto.a.23898 31603610

[34] Muskovic W, Powell JE. DropletQC: improved identification of empty droplets and damaged cells in single-cell RNA-seq data - analysis code. GitHub. https://github.com/powellgenomicslab/dropletQC_paper. 2021. 10.1186/s13059-021-02547-0 PMC8641258 34857027

[35] Muskovic W, Powell JE. DropletQC: improved identification of empty droplets and damaged cells in single-cell RNA-seq data - analysis code. Zenodo. 2021. 10.5281/zenodo.5708997. 10.1186/s13059-021-02547-0 PMC8641258 34857027

[36] Muskovic W, Powell JE. DropletQC: improved identification of empty droplets and damaged cells in single-cell RNA-seq data. GitHub. https://github.com/powellgenomicslab/DropletQC. 2021. 10.1186/s13059-021-02547-0 PMC8641258 34857027

[37] Muskovic W, Powell JE. DropletQC: improved identification of empty droplets and damaged cells in single-cell RNA-seq data. Zenodo. 2021. 10.5281/zenodo.5708994. 10.1186/s13059-021-02547-0 PMC8641258 34857027

