# Supplementary material for: DropletQC: improved identification of empty droplets and damaged cells in single-cell RNA-seq data
Source: Genome Biol. 2021 Dec 2;22:329. doi: 10.1186/s13059-021-02547-0 (PMC8641258; doi:10.1186/s13059-021-02547-0)
Supplement: Supplementary file 1 — Additional file 1. A document containing supplementary figures 1-8 and supplementary tables 1-3 referenced in the main text of the manuscript [file 13059_2021_2547_MOESM1_ESM.docx]

**Supplementary Figures
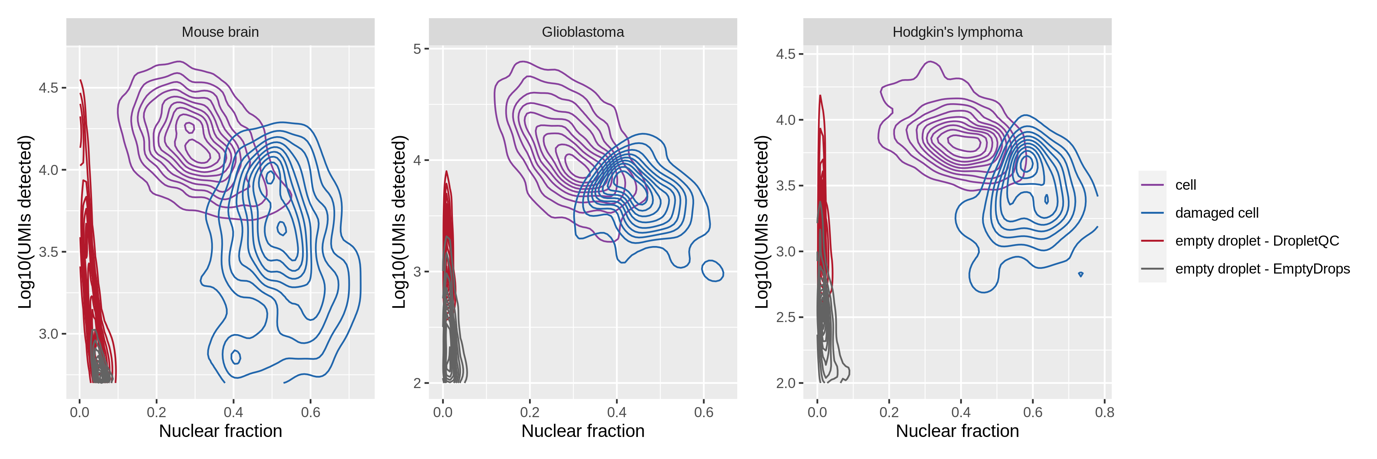
**

**Supplementary Figure 1** | Droplets that are cell free vs those containing intact or damaged cells, exhibit distinct nuclear fraction scores. Two populations of cell free droplets are included; those retained after filtering with EmptyDrops and identified by DropletQC, and droplets excluded by EmptyDrops containing at least 100 UMIs. Cell free droplets are comprised of mostly mature mRNA, and correspondingly have a low nuclear fraction score.

**
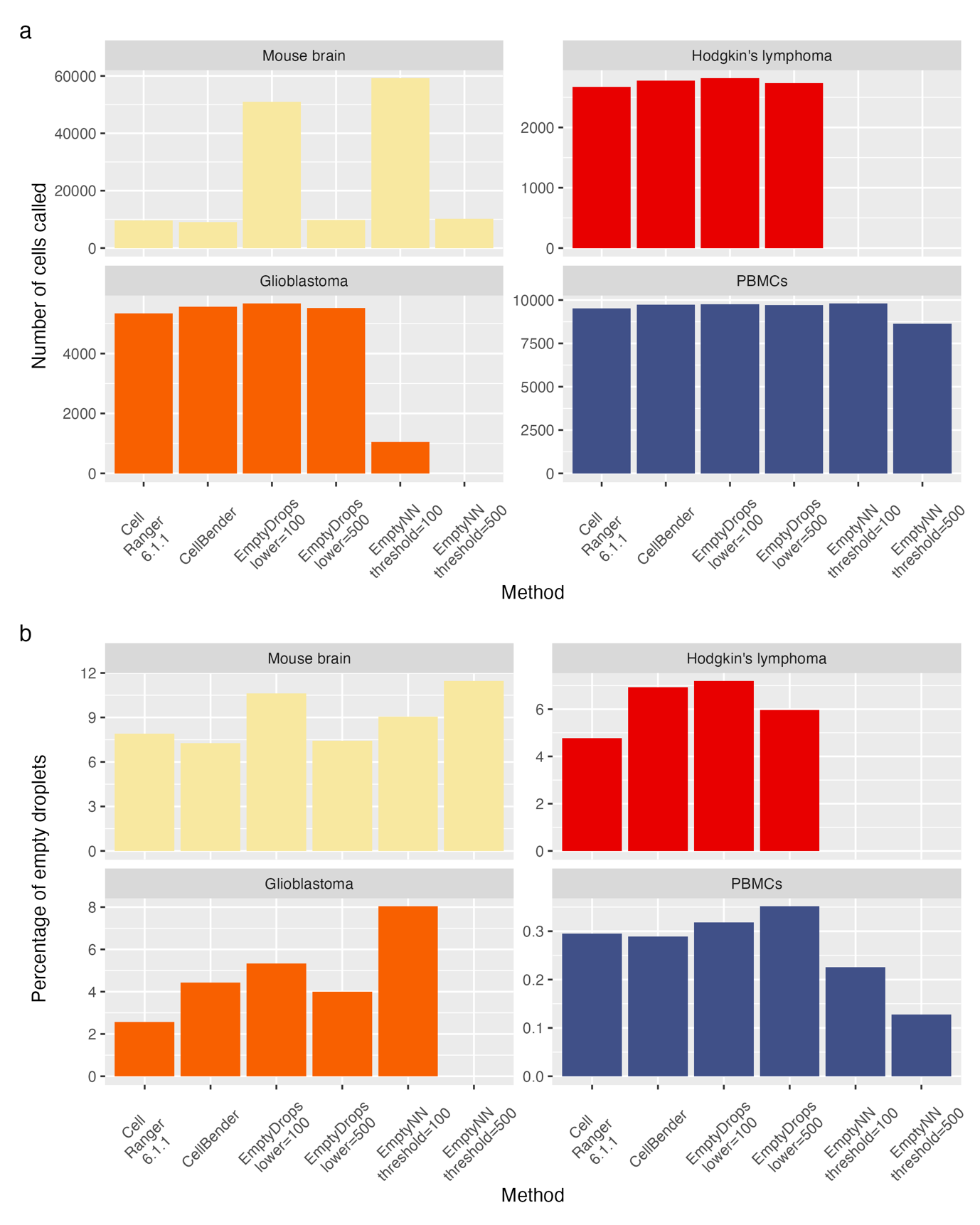
**

**Supplementary Figure 2** | DropletQC identifies cell free droplets missed by other methods. **(a)** The number of cells detected by each method was similar, with EmptyDrops/EmptyNN requiring an increase in the lower/threshold parameter from 100 to 500. EmptyNN failed to detect cells for some samples. **(b)** All methods retained droplets identified by DropletQC as cell free.

**
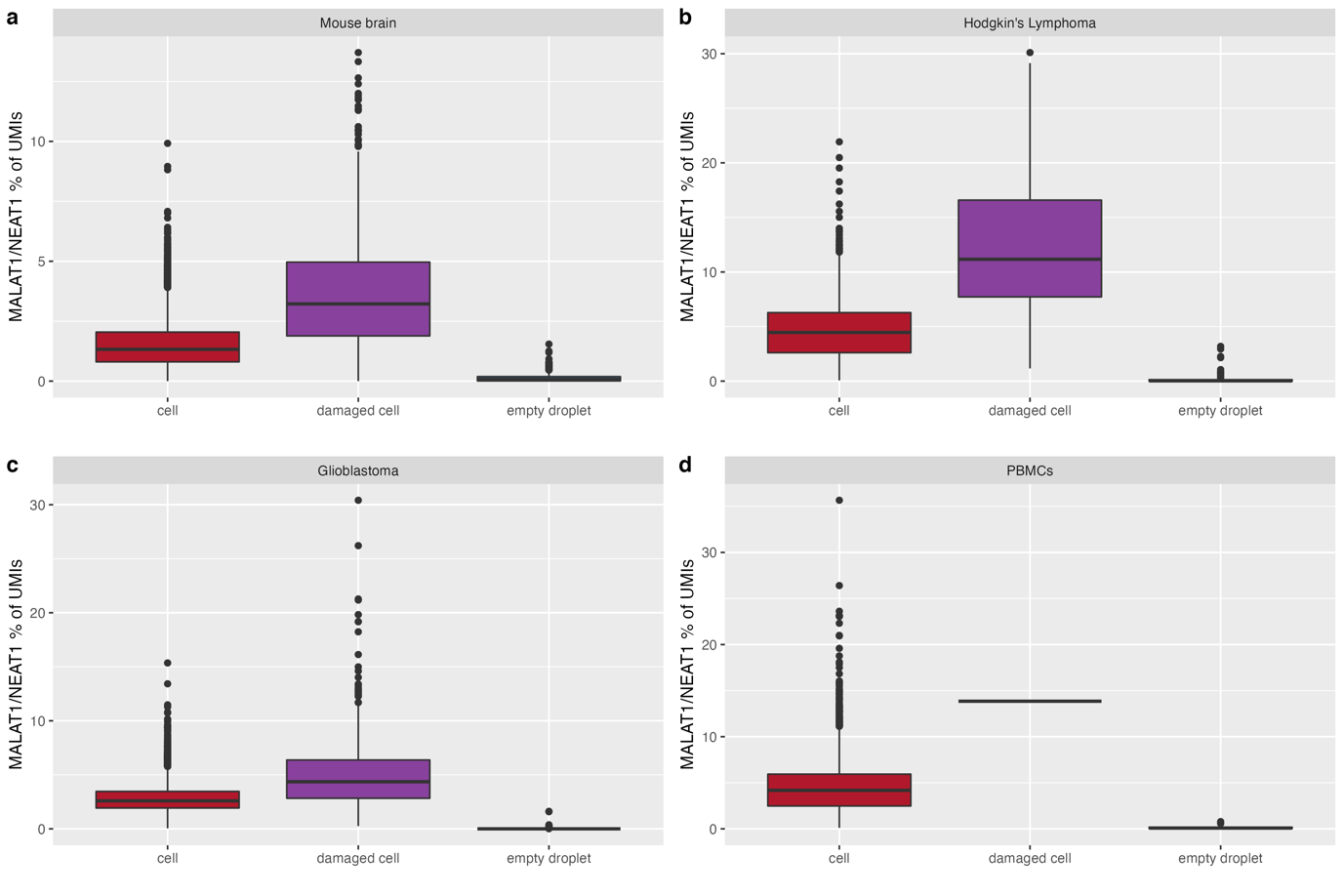
**

**Supplementary Figure 3** | Droplets identified by DropletQC as cell free demonstrate low expression of nuclear-restricted lncRNAs; MALAT1 and NEAT1.


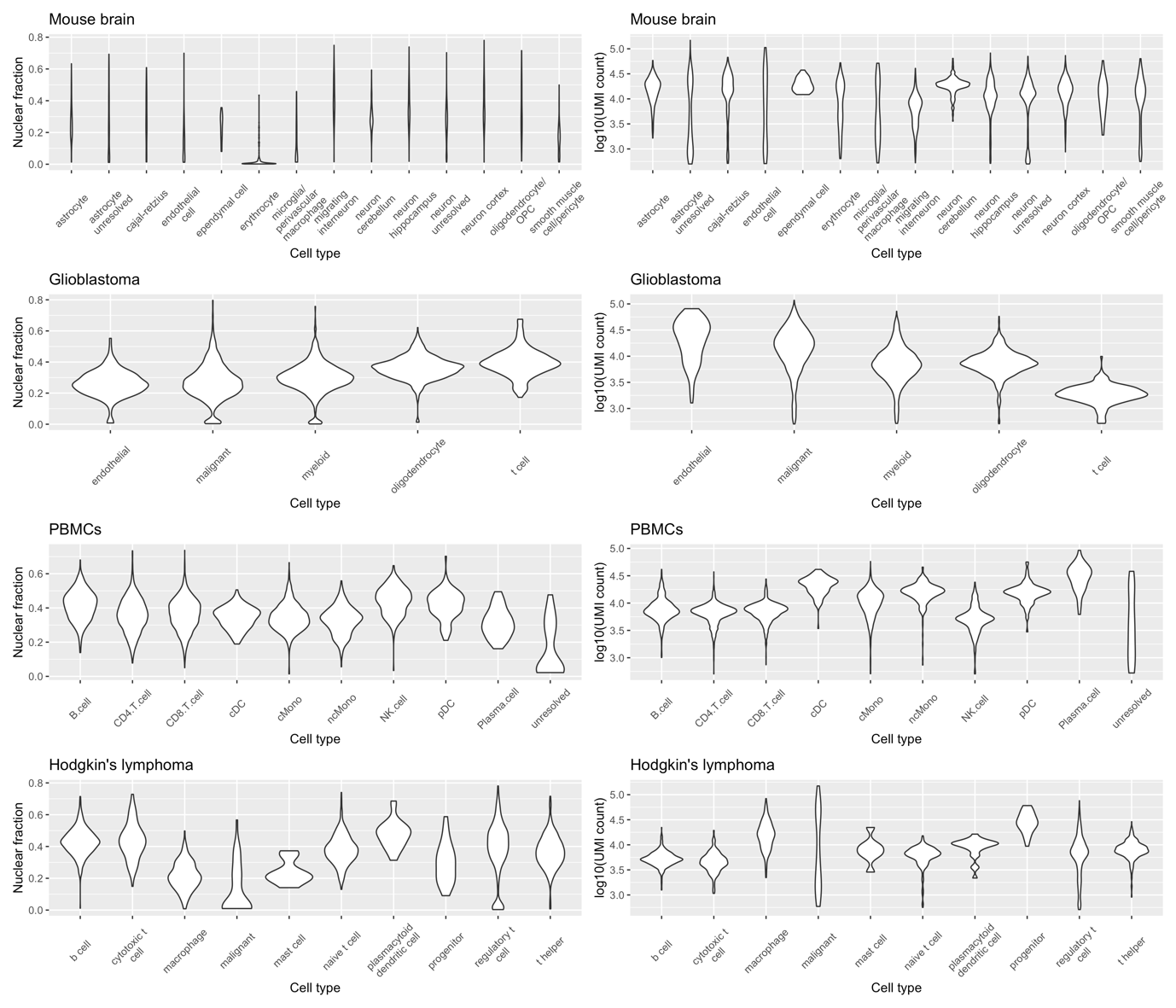


**Supplementary Figure 4** | Different cell types have distinct distributions of nuclear fraction scores and UMI counts.


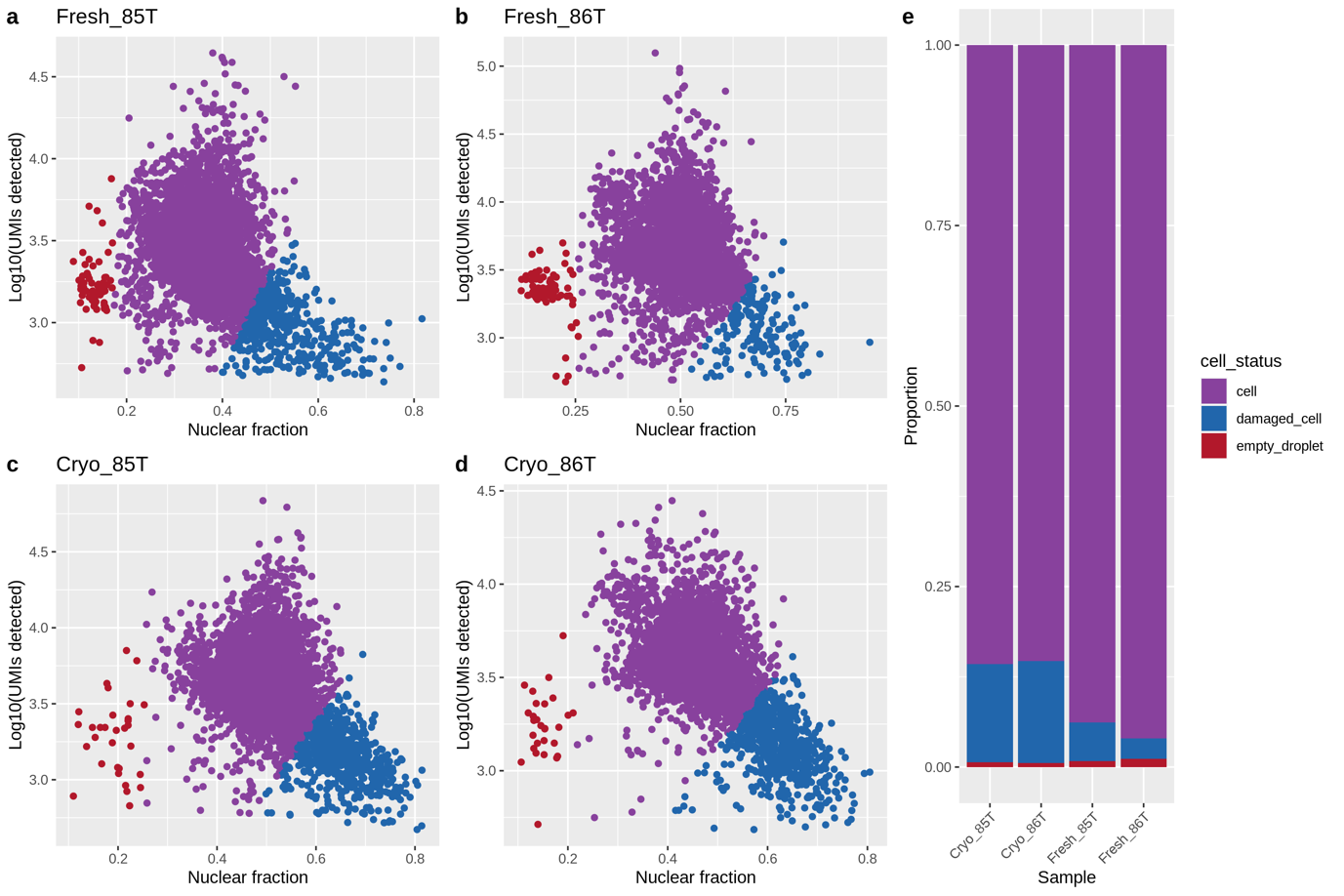


**Supplementary Figure 5 |** DropletQC identifies an increased proportion of damaged cells in cryopreserved microglia samples. **(c-d)** compared to fresh tissue samples **(a-b)**. Total UMI counts (y-axis) and nuclear fraction scores (x-axis) are shown for each cell, with colours representing the status of each cell assigned by DropletQC. The stacked bar chart **(e)** illustrates the proportion of empty droplets and damaged cells for each sample.

*
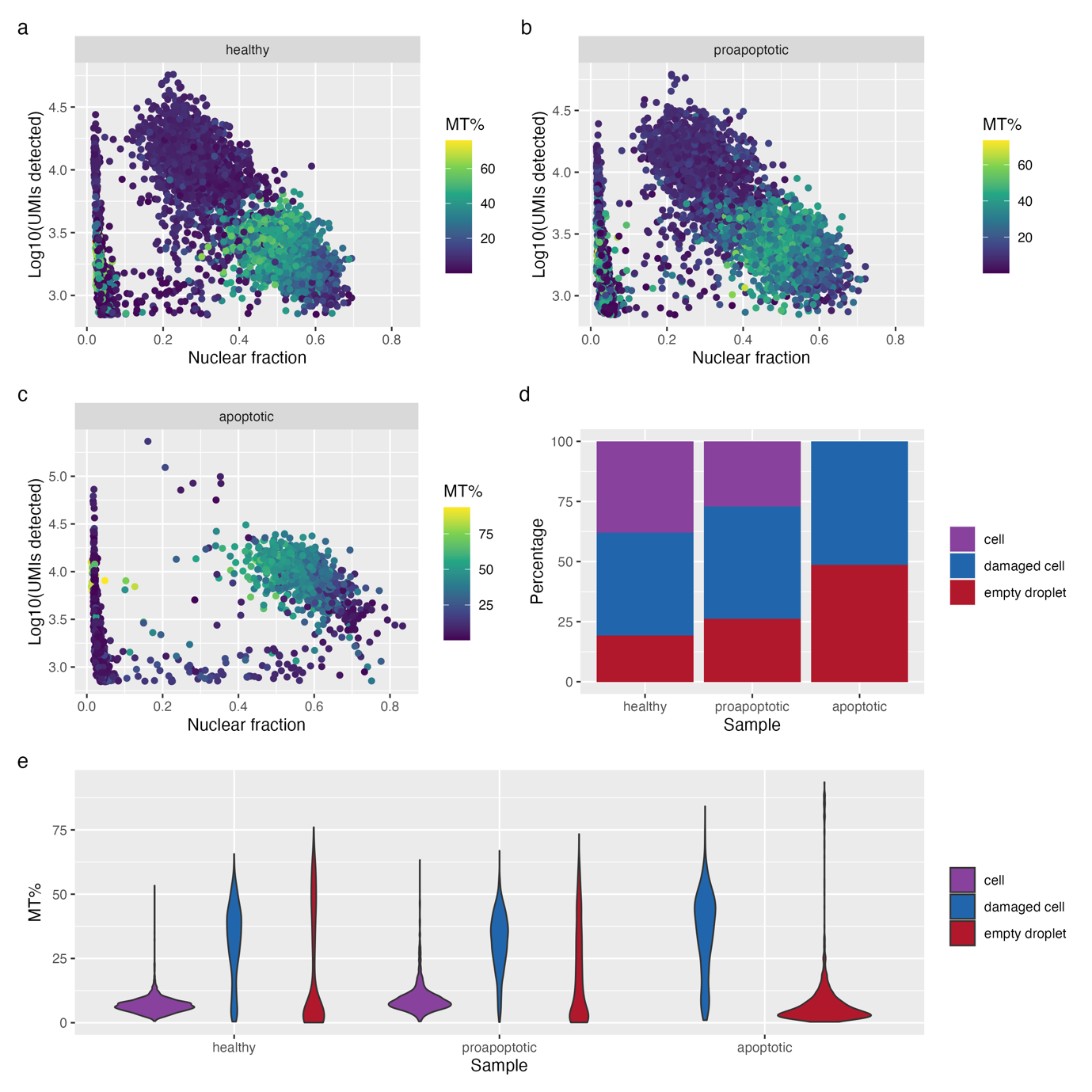
*

**Supplementary Figure 6** **|** DropletQC successfully identified damaged cells in a dataset of HEK293 cells treated with staurosporine. **(a-c)** Total UMI counts (y-axis) and nuclear fraction scores (x-axis) are shown for each cell, with colours representing the percentage of mitochondrial gene content of each cell. **(d)**The proportion of cell free droplets and damaged cells increases as cells progress toward the late-apoptotic state. **(e)** Cells identified by DropletQC as damaged are associated with a higher mitochondrial gene content, a hallmark of damaged and dying cells.


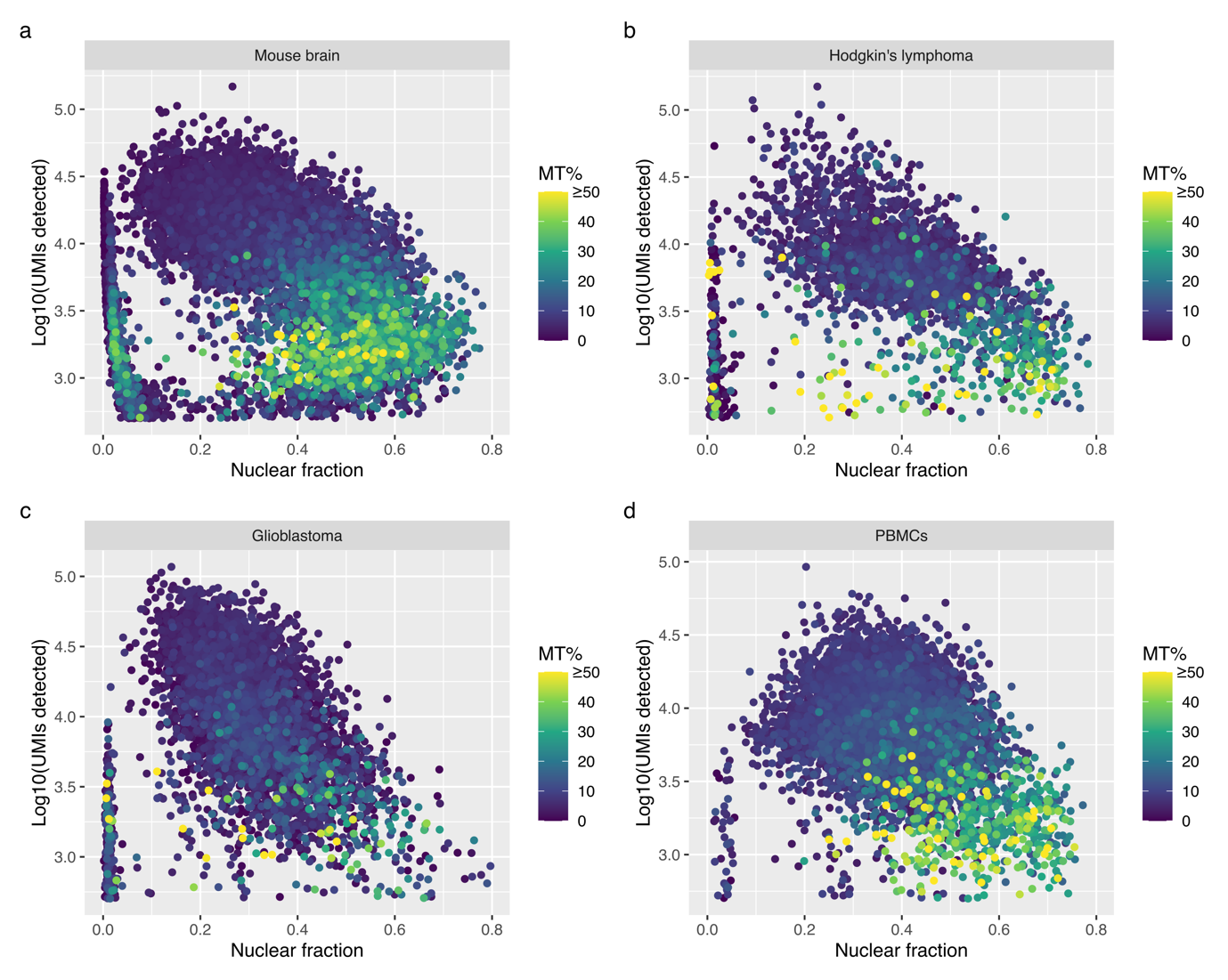


**Supplementary Figure 7** **|** Damaged cells, characterised by a high nuclear fraction and low number of UMIs, are associated with a higher mitochondrial gene content. Total UMI counts (y-axis) and nuclear fraction scores (x-axis) are shown for each cell, with colours representing the percentage of mitochondrial gene content of each cell. Samples were filtered with EmptyDrops to exclude the majority of empty droplets but were not filtered for mitochondrial gene content.

**
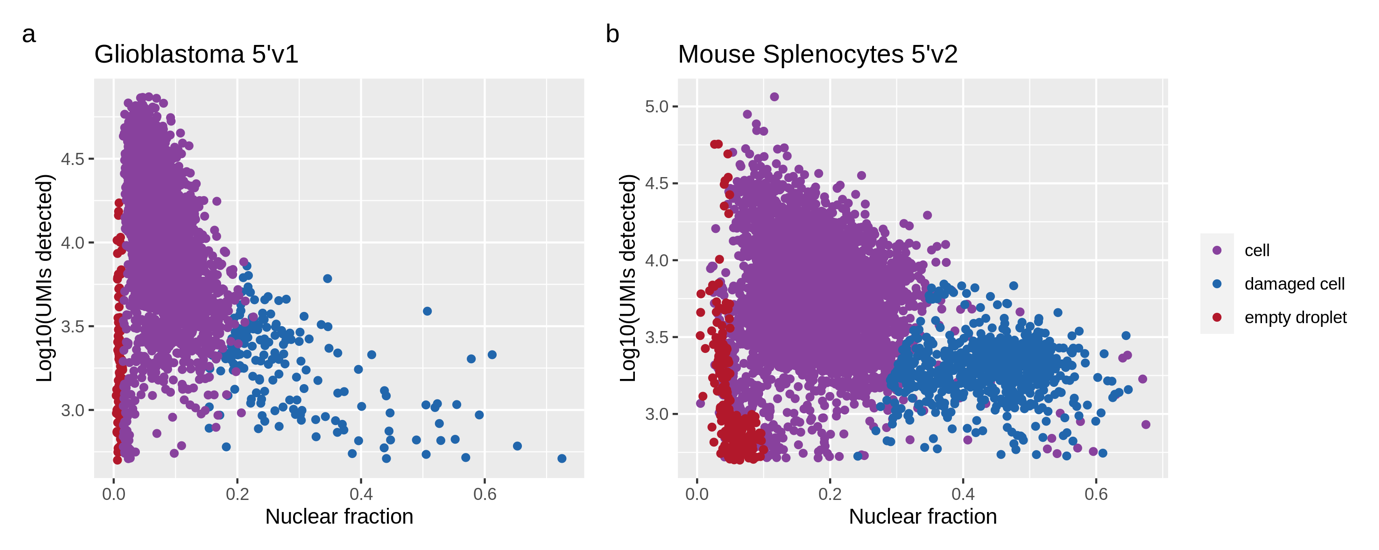
**

**Supplementary Figure 8 |** DropletQC identifies empty droplets and damaged cells in a human glioblastoma **(a)** and mouse splenocyte **(b)** 5' scRNA-seq dataset following filtering with EmptyDrops. Total UMI counts (y-axis) and nuclear fraction scores (x-axis) are shown for each cell, with colours representing the status of each cell assigned by DropletQC. Empty droplets contain less RNA than cells and a higher fraction of cytoplasmic RNA (low nuclear fraction score). Damaged cells contain less RNA than intact cells and a higher proportion of unspliced RNA fragments (high nuclear fraction score).

**Supplementary Tables**

| Sample | Empty droplets | Damaged cells |
| --- | --- | --- |
| Embryonic mouse brain | 9.53% | 14.00% |
| Glioblastoma tumour | 3.99% | 9.77% |
| Peripheral blood mononuclear cells | 0.37% | 0.01% |
| Hodgkin’s lymphoma tumour | 6.04% | 5.16% |

**Supplementary Table 1 |** Summary of the percentage of empty droplets and damaged cells identified in four heterogeneous scRNA-seq datasets after filtering with EmptyDrops with a UMI threshold of 500 and a maximum mitochondrial gene content of 15%. This table quantifies the cell populations presented in Figure 2.

| Sample | Empty droplets | Damaged cells |
| --- | --- | --- |
| Fresh_85T | 0.80% | 5.40% |
| Fresh_86T | 1.15% | 2.84% |
| Cryo_85T | 0.64% | 13.40% |
| Cryo_86T | 0.57% | 14.10% |

**Supplementary Table 2 |** Summary of the percentage of empty droplets and damaged cells identified in four macaque microglia scRNA-seq datasets.

| Sample | Empty droplets | Damaged cells |
| --- | --- | --- |
| Human glioblastoma 5' v1 | 3.13% | 4.42% |
| Mouse splenocytes 5' v2 | 2.42% | 7.64% |

**Supplementary Table 3 |** Summary of the percentage of empty droplets and damaged cells identified in two 5' scRNA-seq datasets following filtering with EmptyDrops.
