# Supplementary material for: DropletQC: improved identification of empty droplets and damaged cells in single-cell RNA-seq data
Source: Genome Biol. 2021 Dec 2;22:329. doi: 10.1186/s13059-021-02547-0 (PMC8641258; doi:10.1186/s13059-021-02547-0)
Supplement: Supplementary file 2 — Additional file 2. Review history [file 13059_2021_2547_MOESM2_ESM.docx]

Review History

**First round of review**

**Reviewer 1**

**Were you able to assess all statistics in the manuscript, including the appropriateness of statistical tests used?**

Yes, and I have assessed the statistics in my report.

**Comments to author:**

Summary The authors present DropletQC - a computational pipeline for scRNA-seq data quality-control. The main achievements the authors describe are: (1) Improved identification of cell-containing droplets relative to the existing gold-standard EmptyDrops, and (2) New ability to distinguish droplets containing intact and damaged cells. DropletQC accomplishes these tasks using a metric called 'nuclear fraction', which describes the relative proportions of cytoplasmic and nuclear RNA in a droplet vis-à-vis (un)spliced RNA proportions. Empty droplets are characterized by low levels of cytoplasmic RNA (low RNA UMIs, low nuclear fraction), droplets containing intact cells have high levels of mixed RNA (high RNA UMIs, intermediate nuclear fraction), and droplets containing damaged/dying cells have low levels of unspliced RNA (low RNA UMIs, high nuclear fraction). To benchmark DropletQC, the authors apply the method to four publicly-available scRNA-seq datasets and showed that their method improves upon EmptyDrops in the task of empty droplet identification (although they should emphasize why this is the case more effectively in the main text — Minor Comment #1). The authors then use DropletQC to identify damaged cells in these data, as well as in scRNA-seq datasets of fresh and cryopreserved macaque brain cells. Correlative analyses demonstrate that accuracy of DropletQC damaged cell classifications (e.g., increase in damaged cells with fresh vs cryopreserved macaque brain samples, decrease in damaged cells for samples not requiring stressful preparation workflows (e.g. PBMCs) vs samples requiring stressful dissociation (e.g., mouse brain)), although I believe the authors can better support these results (Minor Comment #2).

On balance, I believe that the manuscript is well-executed and the 'nuclear fraction' metric will likely become a quality-control gold-standard for future single-cell genomics analysis efforts along the lines of mitochondrial gene content. I also want to give kudos to the authors for writing a very clear and concise manuscript. However, I do not believe that the authors did an adequate job of answering the key (in my opinion) question for single-cell computational methods papers (Major Comment #1) — If I am a reader / potential user, should I use this on my data? Moreover, I have concerns regarding the EmptyDrops comparison (Major Comment #2) and believe that the authors should additionally explore the utility of mitochondrial gene content for identifying damaged cells (Major Comment #3). Assuming these major comments are addressed, however, I strongly suggest that this manuscript is published in Genome Biology.

Major Comments

1. It is very clear that nuclear fraction is a useful quality-control metric for scRNA-seq analysis. However, there are two variables that might influence the utility of nuclear fraction for quality-control - reference transcriptome and scRNA-seq chemistry. For reference transcriptome - is DropletQC limited to species with reference transcriptomes where exonic and intronic regions are well-defined? For scRNA-seq chemistry - does DropletQC perform comparably for 3' and 5' scRNA-seq assays? Authors should either include analyses demonstrating the applicability of these methods across these variables, or provide disclaimers about the anticipated utility/issues with applying DropletQC to such data.

2. Why was the UMI count threshold for EmptyDrops increased from 100 to 500? Could this explain why DropletQC was able to identify empty droplets missed by EmptyDrops?

3. Why were cells with >15% mitochondrial gene content excluded? My understanding of mitochondrial gene content thresholding — i.e., it marks damaged cells leaking cytoplasmic RNA — suggests that high mitochondrial gene content cells and high nuclear fraction cells should be highly overlapping. Does nuclear fraction capture a signature of 'damage' that is not mirrored by mitochondrial gene content? And along this line of thought - have the authors tried including and/or substituting mitochondrial gene content in for nuclear fraction during the GMM step? How does the performance change? If mitochondrial gene content and nuclear fraction are colinear, then it might make even more sense just to use the authors' computational approach without nuclear fraction to avoid increased computation time and to avoid (theoretical) sensitivities to reference transcriptome quality and sequencing chemistry.

Minor Comments

1. The authors do a good job of succinctly describing how nuclear fraction is used to identify damaged cells (Lines 90-94), with the corresponding details in the Methods section. I suggest that the authors do something similar in the previous paragraph for empty droplet estimation (i.e., local minima estimation following kernel density estimation) to give the reader a hint as to why DropletQC outperforms EmptyDrops during this task.

2. Analysis using the macaque dataset where libraries were prepared +/- cryopreservation is well-executed in that it validates the utility of nuclear fraction modeling for damaged cell identification. However, subsequent analysis of the four described datasets showing that the DropletQC-defined damaged cells have some hallmarks of this phenotype (e.g., cell stress markers, high mitochondrial gene content, etc.) would further reinforce the message. This isn't necessary for the manuscript (hence the minor comment), but would be nice.

3. It is difficult to assess overlap between intact and damaged cells in the log10(UMI) x nuclear fraction plots in Fig. 2 and Fig. S2. Perhaps the authors can plot densities instead of points? Or reduce the point size and/or opacity?

4. In Line 60, the authors state, "… cell-free droplets with high ambient RNA concentration and damaged cells are still retained by [EmptyDrops]." This is probably true, but since this is a core argument for justifying the development of DropletQC, the authors should provide at least a citation supporting this claim. Or the authors could give a citation and a 1-2 sentence example of an instance where ambient RNA and/or damaged cells missed by EmptyDrops has caused a concrete issue in data interpretation.

5. Authors should clarify in the discussion that users should apply DropletQC and EmptyDrops in tandem — i.e., initial pruning of cell barcodes using EmptyDrops, then further refinement using DropletQC. I also might be misinterpreting the workflow, so please correct if I am mistaken.

**Reviewer 2**

**Were you able to assess all statistics in the manuscript, including the appropriateness of statistical tests used?**

Yes, and I have assessed the statistics in my report.

**Comments to author:**

The paper introduces DropletQC, a statistical method together with an R package for detecting empty droplets and low-quality cells in scRNA-seq experiments. In brief, the authors suggest using the fraction of nuclear transcripts as a QC metric for detecting low-quality cells, according to the mechanistic picture that damaged and/or partially lysed cells leak a fraction of their cytoplasmic transcripts, resulting in an enrichment of nuclear mRNA. Similarly, the authors demonstrate that empty droplets can be identified as having low nuclear fraction and small UMI count. Using the fraction of nuclear mRNA as a QC metric is not a novel idea; this idea has appeared before as a means to identify low-quality cells (e.g. see [1]), is a commonly extracted QC metric (e.g. see [2]) among other QC metrics. One of the major challenges with scRNA-seq QC is the apparent lack of a "one size fits all" strategy. This issue is fundamental and is deeply rooted in the same biology we wish to explore using scRNA-seq: different cell types exhibit exceedingly diverse phenotypes and every common QC metric (e.g. # of UMI, # of unique genes, # of exonic/intronic alignments, # of mito/ribo genes, etc.), without exception, is strongly correlated with the cell type / cell state.

I have two fundamental issues with this manuscript:

1. The authors have presented the idea of clustering cells in the two-dimensional plane of "fraction of nuclear mRNA" vs. "total UMI count" as a generally-applicable single-cell QC strategy. Both of these metrics are strongly correlated with actual biology rather universally. In particular, nuclear and cytoplasmic RNA abundances are known to be strongly correlated with cell states [3]. As such, a QC method based on this simplistic picture is bound to lead to biased outcomes and worryingly, throw out actual biological signal in the mix of low-quality cells. Even though the authors have run their analysis on different tissue types, the findings are not adequately discussed or validated: are the damaged cells, as flagged by DropletQC, actually damaged (e.g. verified by other means)? is there a significant enrichment of flagged cells in certain cell types / states? if so, is it warranted to flag and remove such cells as being of low-quality?

2. The authors have not benchmarked their method against other existing QC approaches and/or methodologies for detecting empty droplets other than EmptyDrops, which is often taken as a baseline method. In summary, I do not find the manuscript to represent a significant advance over previously published studies, or of outstanding interest to a broad audience of biologists in its present form.

[1] Tucker, Nathan R., et al. "Transcriptional and cellular diversity of the human heart." Circulation 142.5 (2020): 466-482.

[2] Tian, Luyi, et al. "scPipe: A flexible R/Bioconductor preprocessing pipeline for single-cell RNA-sequencing data." PLoS computational biology 14.8 (2018): e1006361.

[3] Abdelmoez, Mahmoud N., et al. "SINC-seq: correlation of transient gene expressions between nucleus and cytoplasm reflects single-cell physiology." Genome biology 19.1 (2018): 1-11.

**Reviewer 3**

**Were you able to assess all statistics in the manuscript, including the appropriateness of statistical tests used?**

Yes, and I have assessed the statistics in my report.

**Comments to author:**

Summary The authors present DropletQC, a method developed to discriminate between empty droplets, droplets with damaged cells, and droplets with intact cells in the context of single cell RNA sequencing (scRNA-seq) data analysis. To do so they utilize a QC metric called "nuclear fraction", which is the fraction of reads that map to regions of the genome annotated as introns. The method is publicly available as an R package, and the author apply it to four scRNA-seq data sets and discuss results.

General comments

A tool to better assess droplet quality and improve the status quo of scRNA-seq data analysis would be of great interest, and the idea to use pre/mature RNA signatures to stratify the RNA content of droplets is compelling. However, I have three major concerns about the manuscript:

1) Method assessment. The authors illustrate the annotation results of their method in Figure 2, but it remains unclear whether droplets annotated as "empty" or "damaged" are indeed empty or damaged. Without providing some additional support for the derived annotations it is not clear how to evaluate and interpret these results.

2) Cell-type specificity. The authors show differences in nuclear fraction across cell-types in Supplementary Figure 1, but it remains unclear whether these differences are due to different sensitivities of respective cells to library construction, or if they represent "physiological" differences. It seems that only in the latter case a cell-type-specific analysis would be warranted. This is of interest because high-quality cell-type annotation often remains non-trivial, as automated cell-type annotation typically needs to be complemented by domain experts. This leads to differences in annotation quality that, in turn, would likely affect DropletQC's performance.

3) Comparison with status quo. It is unclear if additional empty droplets identified by DropletQC (and damaged droplets, also) would have been identified by EmptyDrops with different parameter settings. It would be important to show that droplets additionally flagged by DropletQC are truly complementary and not easily identifiable by current methods.

Specific comments

L 71-73: The authors write that droplets that contain only ambient RNA have low nuclear fraction, compared with droplets containing (intact?) cells. While intuitive, the authors should be clear if this a hypothesis or known (and in that case provide a citation).

L 162 - 194: Is the procedure described different from standard mixture model fitting? If so, it would be helpful to point out where and how it boosts performance. If not, I would suggest removing the procedure and either cite the original work or a software package for Gaussian mixture models, like mclust for example.

**Authors Response**

**Point-by-point responses to the reviewers’ comments:**

Reviewer #1: Summary

The authors present DropletQC - a computational pipeline for scRNA-seq data quality-control. The main achievements the authors describe are: (1) Improved identification of cell-containing droplets relative to the existing gold-standard EmptyDrops, and (2) New ability to distinguish droplets containing intact and damaged cells. DropletQC accomplishes these tasks using a metric called 'nuclear fraction', which describes the relative proportions of cytoplasmic and nuclear RNA in a droplet vis-à-vis (un)spliced RNA proportions. Empty droplets are characterized by low levels of cytoplasmic RNA (low RNA UMIs, low nuclear fraction), droplets containing intact cells have high levels of mixed RNA (high RNA UMIs, intermediate nuclear fraction), and droplets containing damaged/dying cells have low levels of unspliced RNA (low RNA UMIs, high nuclear fraction).

To benchmark DropletQC, the authors apply the method to four publicly-available scRNA-seq datasets and showed that their method improves upon EmptyDrops in the task of empty droplet identification (although they should emphasize why this is the case more effectively in the main text — Minor Comment #1). The authors then use DropletQC to identify damaged cells in these data, as well as in scRNA-seq datasets of fresh and cryopreserved macaque brain cells. Correlative analyses demonstrate that accuracy of DropletQC damaged cell classifications (e.g., increase in damaged cells with fresh vs cryopreserved macaque brain samples, decrease in damaged cells for samples not requiring stressful preparation workflows (e.g. PBMCs) vs samples requiring stressful dissociation (e.g., mouse brain)), although I believe the authors can better support these results (Minor Comment #2).

On balance, I believe that the manuscript is well-executed and the 'nuclear fraction' metric will likely become a quality-control gold-standard for future single-cell genomics analysis efforts along the lines of mitochondrial gene content. I also want to give kudos to the authors for writing a very clear and concise manuscript. However, I do not believe that the authors did an adequate job of answering the key (in my opinion) question for single-cell computational methods papers (Major Comment #1) — If I am a reader / potential user, should I use this on my data? Moreover, I have concerns regarding the EmptyDrops comparison (Major Comment #2) and believe that the authors should additionally explore the utility of mitochondrial gene content for identifying damaged cells (Major Comment #3). Assuming these major comments are addressed, however, I strongly suggest that this manuscript is published in Genome Biology.

*We thank reviewer #1 for this detailed summary and insightful and helpful comments on our work. We have addressed each of these below.*

Major Comments

1. It is very clear that nuclear fraction is a useful quality-control metric for scRNA-seq analysis. However, there are two variables that might influence the utility of nuclear fraction for quality-control - reference transcriptome and scRNA-seq chemistry. For reference transcriptome - is DropletQC limited to species with reference transcriptomes where exonic and intronic regions are well-defined? For scRNA-seq chemistry - does DropletQC perform comparably for 3' and 5' scRNA-seq assays? Authors should either include analyses demonstrating the applicability of these methods across these variables, or provide disclaimers about the anticipated utility/issues with applying DropletQC to such data.

*We thank reviewer #1 for raising these points. It is correct that calculating the nuclear fraction would be challenging in instances where there are poorly annotated intron and exon coordinates. We have highlighted this limitation in the revised discussion, noting that the accuracy of the nuclear fraction calculation will indeed depend on the quality of the transcriptome. We have also included a reference to the “nuclear_fraction_annotation” function described in the “Nuclear fraction calculation” section of the methods, which provides an option for use a user-supplied gene annotation, which can be easily updated as the quality of the species’ gene annotation is improved. We also note that such instances will be rare in single cell studies, where the vast majority are conducted on either human or model organism samples.*

*We did not anticipate any challenges with using data generated from 5’ assays, however it was an oversight to not include analysis of this data in our manuscript - thank you for highlighting this! We have expanded our analysis to include data from two 5' scRNA-seq datasets; a human glioblastoma tumour (5' v1 chemistry) and a mouse splenocytes dataset (5' v2 chemistry). The results have been included in the manuscript and presented in Figure S7 and Table S3. They demonstrate DropletQC works well with 5' assays, identifying both empty droplets and damaged cells.*

2. Why was the UMI count threshold for EmptyDrops increased from 100 to 500? Could this explain why DropletQC was able to identify empty droplets missed by EmptyDrops?

*We apologize for not adequately explaining this parameter choice in the originally submitted manuscript. We have updated the methods to include a note on this and have also included the results of running EmptryDrops with the default threshold if 100, along with additional QC methods, in response to a request from reviewer #2, presented in Figure S2 and expanded on in our response to reviewer #2 below. Briefly, the default UMI threshold is suitable for many scRNA-seq datasets, however the datasets included in Figure 2 have an above average sequencing depth. Using the default parameter results in significantly poorer performance of EmptryDrops, and so we had increased this to the more suitable value of 500 for a fair comparison against DropletQC.*

3. Why were cells with >15% mitochondrial gene content excluded? My understanding of mitochondrial gene content thresholding — i.e., it marks damaged cells leaking cytoplasmic RNA — suggests that high mitochondrial gene content cells and high nuclear fraction cells should be highly overlapping. Does nuclear fraction capture a signature of 'damage' that is not mirrored by mitochondrial gene content?

And along this line of thought - have the authors tried including and/or substituting mitochondrial gene content in for nuclear fraction during the GMM step? How does the performance change? If mitochondrial gene content and nuclear fraction are colinear, then it might make even more sense just to use the authors' computational approach without nuclear fraction to avoid increased computation time and to avoid (theoretical) sensitivities to reference transcriptome quality and sequencing chemistry.

*We thank the reviewer for pointing out that this was not adequately explained in the manuscript. We have now expanded the description of the mitochondrial gene content (MT%) filtering in the methods section. Cells were filtered based on MT% to be in line with current practices, and in order to present a fair comparison with existing QC approaches. We expand on this in our response to reviewer #2’s first comment below, where we have included new analysis to show cells identified by DropletQC are indeed damaged and associated with a higher MT% (Figure S5, Figure S6). The relationship is not collinear, with damaged and dying cells exhibiting a broad range of MT% (Fig. S5 a-c, e).*

Minor Comments

1. The authors do a good job of succinctly describing how nuclear fraction is used to identify damaged cells (Lines 90-94), with the corresponding details in the Methods section. I suggest that the authors do something similar in the previous paragraph for empty droplet estimation (i.e., local minima estimation following kernel density estimation) to give the reader a hint as to why DropletQC outperforms EmptyDrops during this task.

*We have updated the relevant paragraph to provide a brief description of how DropletQC identifies cell free droplets.*

2. Analysis using the macaque dataset where libraries were prepared +/- cryopreservation is well-executed in that it validates the utility of nuclear fraction modeling for damaged cell identification. However, subsequent analysis of the four described datasets showing that the DropletQC-defined damaged cells have some hallmarks of this phenotype (e.g., cell stress markers, high mitochondrial gene content, etc.) would further reinforce the message. This isn't necessary for the manuscript (hence the minor comment), but would be nice.

*We appreciate this suggestion and have performed additional analysis to validate that the droplets identified by DropletQC are indeed damaged and display a higher MT%, a hallmark of this phenotype as reviewer #1 points out. This is outlined in our response to reviewer #2’s first comment below also.*

*To demonstrate that cells flagged by DropletQC are indeed damaged we felt a positive control was required. Accordingly, we have performed additional analysis, running DropletQC on HEK293 cells treated with staurosporine and captured in a healthy, pro- and late-apoptotic state - with cells FACS sorted based on apoptotic markers, providing orthogonal information as to their state. We show that the proportion of damaged cells identified by DropletQC increases as cells proceed towards the apoptotic state (Figure S5 a-d) and that damaged cells are associated with increased mitochondrial gene content, a hallmark of damaged and dying cells (Figure S5 e). We additionally show that cells identified as damaged in the four main datasets (Figure 2) are similarly associated with increased mitochondrial gene content (Figure S6).*

3. It is difficult to assess overlap between intact and damaged cells in the log10(UMI) x nuclear fraction plots in Fig. 2 and Fig. S2. Perhaps the authors can plot densities instead of points? Or reduce the point size and/or opacity?

*We thank reviewer #1 for this suggestion and have included a density plot of these samples in Figure S1 to provide an alternative illustration of the different populations of droplets.*

4. In Line 60, the authors state, "… cell-free droplets with high ambient RNA concentration and damaged cells are still retained by [EmptyDrops]." This is probably true, but since this is a core argument for justifying the development of DropletQC, the authors should provide at least a citation supporting this claim. Or the authors could give a citation and a 1-2 sentence example of an instance where ambient RNA and/or damaged cells missed by EmptyDrops has caused a concrete issue in data interpretation.

*We thank reviewer #1 for highlighting this point should be better supported in the manuscript. We now demonstrate this empirically in Figure S1. As many captured droplets are cell-free, we have included them in the figure, showing they contain mRNA and hence have a very low nuclear fraction.*

5. Authors should clarify in the discussion that users should apply DropletQC and EmptyDrops in tandem — i.e., initial pruning of cell barcodes using EmptyDrops, then further refinement using DropletQC. I also might be misinterpreting the workflow, so please correct if I am mistaken.

*We have followed this recommendation and incorporated an additional sentence in the discussion section to this effect.*

Reviewer #2:

The paper introduces DropletQC, a statistical method together with an R package for detecting empty droplets and low-quality cells in scRNA-seq experiments. In brief, the authors suggest using the fraction of nuclear transcripts as a QC metric for detecting low-quality cells, according to the mechanistic picture that damaged and/or partially lysed cells leak a fraction of their cytoplasmic transcripts, resulting in an enrichment of nuclear mRNA. Similarly, the authors demonstrate that empty droplets can be identified as having low nuclear fraction and small UMI count.

Using the fraction of nuclear mRNA as a QC metric is not a novel idea; this idea has appeared before as a means to identify low-quality cells (e.g. see [1]), is a commonly extracted QC metric (e.g. see [2]) among other QC metrics.

One of the major challenges with scRNA-seq QC is the apparent lack of a "one size fits all" strategy. This issue is fundamental and is deeply rooted in the same biology we wish to explore using scRNA-seq: different cell types exhibit exceedingly diverse phenotypes and every common QC metric (e.g. # of UMI, # of unique genes, # of exonic/intronic alignments, # of mito/ribo genes, etc.), without exception, is strongly correlated with the cell type / cell state.

*We would like to thank reviewer #2 for taking the time to review our work and providing helpful comments and suggestions which we have addressed individually below. We agree that other work has previously assessed the percentage of reads that align to different genomic regions and thank reviewer #2 for the provided references. However, we note that while similar information may be obtained through other tools, such as software packages for RNA velocity analysis, we believe our manuscript is the first to demonstrate how this information can be usefully interpreted for quality control of scRNA-seq data. Our approach identifies both contaminating cell free droplets and damaged cells. We further note that our analysis of publicly available datasets demonstrates existing QC pipelines may benefit from the use of DropletQC.*

I have two fundamental issues with this manuscript:

1. The authors have presented the idea of clustering cells in the two-dimensional plane of "fraction of nuclear mRNA" vs. "total UMI count" as a generally-applicable single-cell QC strategy. Both of these metrics are strongly correlated with actual biology rather universally. In particular, nuclear and cytoplasmic RNA abundances are known to be strongly correlated with cell states [3]. As such, a QC method based on this simplistic picture is bound to lead to biased outcomes and worryingly, throw out actual biological signal in the mix of low-quality cells. Even though the authors have run their analysis on different tissue types, the findings are not adequately discussed or validated: are the damaged cells, as flagged by DropletQC, actually damaged (e.g. verified by other means)? is there a significant enrichment of flagged cells in certain cell types / states? if so, is it warranted to flag and remove such cells as being of low-quality?

*We thank reviewer #2 for raising the two points above; that the number of UMIs and nuclear fraction may be correlated with underlying biology, and that we have not adequately validated that the cells flagged by DropletQC are indeed damaged. We address each of these points individually below.*

*We have clarified in the manuscript how DropletQC flags damaged cells. As reviewer #2 highlights, and as we show in Figure S3, the distributions for the number of UMIs detected and nuclear fraction scores are often distinct for different cell types. For this reason, DropletQC assesses these distributions separately for each cell type. We have clarified this in the main body of the manuscript.*

*We thank reviewer #2 for raising the point that additional evidence is required to demonstrate cells flagged by DropletQC are indeed damaged. To do this we felt a positive control was required. Accordingly, we have performed additional analysis, running DropletQC on HEK293 cells treated with staurosporine and captured in a healthy, pro- and late-apoptotic state - with cells FACS sorted based on apoptotic markers, providing orthogonal information as to their state. We show that the proportion of damaged cells identified by DropletQC increases as cells proceed towards the apoptotic state (Figure S5 a-d) and that damaged cells are associated with increased mitochondrial gene content, a hallmark of damaged and dying cells (Figure S5 e). We additionally show that cells identified as damaged in the four main datasets (Figure 2) are similarly associated with increased mitochondrial gene content (Figure S6).*

*In addition to our responses to the two points above, we address an overarching concern from reviewer #2: that DropletQC may inadvertently remove cells that are biologically interesting. As in many cases damaged cells will be of interest, DropletQC does not remove any cells. DropletQC creates an annotation that can be retained as metadata for downstream analyses where cell status (intact or damaged) may be easily confounded with the biology of interest. This point is further addressed in the discussion section of the manuscript.*

2. The authors have not benchmarked their method against other existing QC approaches and/or methodologies for detecting empty droplets other than EmptyDrops, which is often taken as a baseline method.

*The point raised by reviewer #2, that DropletQC should be compared against additional existing QC approaches, is well taken. We have addressed this by expanding our analysis to compare several tools, including the latest version of the widely-used 10x Genomics Cell Ranger software, and two recently published methods; EmptyNN and CellBender. The results of this analysis are updated in the main text and in Figure S2, showing that all of the tested methods, for all samples, retain cell free droplets which are identified by DropletQC. We further demonstrate that only DropletQC is able to identify damaged cells in addition to the empty droplets.*

In summary, I do not find the manuscript to represent a significant advance over previously published studies, or of outstanding interest to a broad audience of biologists in its present form.

*We thank reviewer #2 for their constructive comments and criticism. We think that we have addressed the points they have raised, which have strengthened the manuscript, such that it will be of broader interest to the single cell community and the readership of Genome Biology.*

[1] Tucker, Nathan R., et al. "Transcriptional and cellular diversity of the human heart." Circulation 142.5 (2020): 466-482.

[2] Tian, Luyi, et al. "scPipe: A flexible R/Bioconductor preprocessing pipeline for single-cell RNA-sequencing data." PLoS computational biology 14.8 (2018): e1006361.

[3] Abdelmoez, Mahmoud N., et al. "SINC-seq: correlation of transient gene expressions between nucleus and cytoplasm reflects single-cell physiology." Genome biology 19.1 (2018): 1-11.

Reviewer #3: Summary

The authors present DropletQC, a method developed to discriminate between empty droplets, droplets with damaged cells, and droplets with intact cells in the context of single cell RNA sequencing (scRNA-seq) data analysis. To do so they utilize a QC metric called "nuclear fraction", which is the fraction of reads that map to regions of the genome annotated as introns. The method is publicly available as an R package, and the author apply it to four scRNA-seq data sets and discuss results.

*We thank reviewer #3 for this summary of the manuscript and address their specific comments below.*

General comments

A tool to better assess droplet quality and improve the status quo of scRNA-seq data analysis would be of great interest, and the idea to use pre/mature RNA signatures to stratify the RNA content of droplets is compelling. However, I have three major concerns about the manuscript:

1) Method assessment. The authors illustrate the annotation results of their method in Figure 2, but it remains unclear whether droplets annotated as "empty" or "damaged" are indeed empty or damaged. Without providing some additional support for the derived annotations it is not clear how to evaluate and interpret these results.

*We thank reviewer #3 for their comment that droplets flagged by DropletQC as cell free or damaged are not sufficiently validated. We have addressed the point regarding cell free droplets by including Figure S1, where we show that cell free droplets flagged by DropletQC have the same properties (no nuclear pre-mRNA and a low number of UMIs detected) as droplets containing only ambient RNA. We have addressed the second point regarding validation of cells DropletQC flags as damaged, by performing additional analysis of HEK293 cells treated with staurosporine, described in detailed in our response to reviewer #2’s first comment above and presented in Figure S5. We have also included evidence that cells identified as damaged are associated with increased mitochondrial gene content (Figure S5a-c,e and Figure S6), a hallmark of damaged and dying cells. We think that together, these additional findings sufficiently support our claim that DropletQC is a useful tool for identifying cell free droplets and damaged cells in scRNA-seq datasets.*

2) Cell-type specificity. The authors show differences in nuclear fraction across cell-types in Supplementary Figure 1, but it remains unclear whether these differences are due to different sensitivities of respective cells to library construction, or if they represent "physiological" differences. It seems that only in the latter case a cell-type-specific analysis would be warranted. This is of interest because high-quality cell-type annotation often remains non-trivial, as automated cell-type annotation typically needs to be complemented by domain experts. This leads to differences in annotation quality that, in turn, would likely affect DropletQC's performance.

*Reviewer #3’s comments on cell type annotation are appreciated. We have updated the discussion to highlight that care should be taken that cell types are accurately annotated to avoid confounding distinct cell types with damaged vs intact cells. We agree cell type annotation is non-trivial, however is an essential step in the analysis of scRNA-seq data. We point out DropletQC may assist in identifying cell free droplets and damaged cells that could otherwise be misinterpreted as representing a distinct cell type.*

3) Comparison with status quo. It is unclear if additional empty droplets identified by DropletQC (and damaged droplets, also) would have been identified by EmptyDrops with different parameter settings. It would be important to show that droplets additionally flagged by DropletQC are truly complementary and not easily identifiable by current methods.

*We thank reviewer #3 for their comment that DropletQC should be compared against additional existing methods. We have expanded our analysis to include additional methods. These results are presented in Figure S2 and described in the manuscript. We show that for all samples using the methods tested, cell free droplets are retained which are subsequently identified by DropletQC, with the droplets containing damaged cells only identified by our method. In this analysis we also address reviewer #3’s comment on the parameter settings for EmptyDrops, an issue that was also raised by reviewer #1. We show that EmptyDrops’ default UMI threshold of 100 was not appropriate for the tested datasets, which was why we initially changed this parameter. We have included the results of running all of the tested methods with their default settings.*

Specific comments

L 71-73: The authors write that droplets that contain only ambient RNA have low nuclear fraction, compared with droplets containing (intact?) cells. While intuitive, the authors should be clear if this a hypothesis or known (and in that case provide a citation).

*We have addressed this comment with our inclusion of Figure S1, where we show empirically that droplets flagged by DropletQC as cell free have the same nuclear fraction as droplets containing only ambient (cytoplasmic) RNA.*

L 162 - 194: Is the procedure described different from standard mixture model fitting? If so, it would be helpful to point out where and how it boosts performance. If not, I would suggest removing the procedure and either cite the original work or a software package for Gaussian mixture models, like mclust for example.

*We thank reviewer #3 for picking up on this. The reference to mclust was present in the ‘References’ section but the link to the citation in the text was lost during editing. This has now been fixed.*

**Second round of review**

**Reviewer 1**

I have no additional comments on the revised manuscript and strongly recommend its publication in Genome Biology.

**Reviewer 3**

The manuscript has benefitted from the revision and the authors have addressed my concerns partially. Here are my comments and remaining concerns:

* While I follow the authors' argument about damaged cells and their Supplemental Figure S5, I'm not convinced about DropletQC's performance for calling cell-free droplets. In their response to my concerns the authors mention Figure S1, but it is unclear how this addresses the concern that empty droplets called by DropletQC are indeed empty. Also, it would be helpful to show that DropletQC, when used for calling empty droplets, indeed identifies many/most empty droplets present. In their comparisons with other methods however, it is not clear if empty droplets called by DropletQC mostly overlap those called by other methods. It would be straight forward, for example, to quantify overlap (and differences, should they exist), for instance in the context of supplemental figure S2.

The R package overall works well and the documentation is helpful. On one of our datasets, however, with standard parameters few empty droplets were identified and many of the droplets passed had really low library size. In practice, it therefore seems likely we would have used an other/additional method for empty droplet calling and DropletQC perhaps for identifying damaged cells. This observation prompted an effort to reproduce supplemental figure S2; however contrary to the authors' statement that "All of the code used to produce the analyses and figures presented in the manuscript, along with links to individual datasets, are available through GitHub at https://github.com/powellgenomicslab/DropletQC_paper.", this repository was not visible/accessible on GitHub. While most likely an oversight, the code appears currently not to be available and this diminishes my confidence in the manuscript and rises questions about the reproducibility of reported findings.

* The space spent on describing a standard procedure for fitting a mixture model still seems excessive, especially since non of the equations are instrumental to the approach, nor are they referred to anywhere else in the manuscript.

**Authors Response**

**Point-by-point responses to the reviewers’ comments:**

*Referee 3 raised the concern that droplets identified by DropletQC as cell-free are not sufficiently validated to be empty. To address this concern, we have performed additional analysis, quantifying the expression of two abundant nuclear-restricted lncRNAs; MALAT1 and NEAT1, in the three populations of droplets identified by DropletQC; empty droplets, droplets containing damaged cells and droplets containing intact cells. The results of this analysis are now included in supplementary figure 3 and are referenced in the manuscript text. These results demonstrate that droplets identified by DropletQC as cell-free contain significantly lower expression of these lncRNAs, and hence are unlikely to contain a cell nucleus and are correctly identified as empty.*

*Referee 3 requested an additional comparison examining the overlap between droplets identified by DropletQC and other tools such as EmptyDrops. Unfortunately, this is not possible, as DropletQC is run after most cell barcodes have been excluded. This is stated in the main text of the manuscript and is necessary, as an accurate nuclear fraction cannot be calculated for droplets with very few UMIs detected.*

*Referee 3 raised an additional concern that they could not access the analysis code in the referenced GitHub repository. We have now ensured the repository is publicly viewable and have additionally created a DOI-assigned copy of the code on zenodo.*
